# ﻿Two new species of *Danaceothrix* Majer, 1989 (Coleoptera, Dasytidae) from Xizang, China

**DOI:** 10.3897/zookeys.1241.147383

**Published:** 2025-06-18

**Authors:** Jialin Miao, Haoyu Liu, Junbo Tong, Xingke Yang, Yuxia Yang

**Affiliations:** 1 Key Laboratory of Zoological Systematics and Application, School of Life Sciences, Hebei University, Baoding 071002, China; 2 Hebei Basic Science Center for Biotic Interaction, Hebei University, Baoding 071002, China; 3 Key Laboratory of Zoological Systematics and Evolution, Institute of Zoology, Chinese Academy of Sciences, Beijing 100101, China

**Keywords:** Alpha taxonomy, dasytid beetles, Himalayas, identification key, morphology

## Abstract

Two new dasytid species of the genus *Danaceothrix* Majer, 1989 are discovered from Xizang, China and described under the names of *D.medogense***sp. nov.** and *D.xizangense***sp. nov.** They are illustrated with habitus, ultimate abdominal tergite and sternite, and genitalia of both sexes. The generic diagnosis is updated. A key for identification and a distribution map of all *Danaceothrix* species are provided.

## ﻿Introduction

The genus *Danaceothrix* Majer, 1989 is currently classified within the subfamily Chaetomalachiinae Majer, 1987 of the beetle family Dasytidae (Majer 1996; Mayor 2007). Adults of *Danaceothrix* can be easily distinguished from all other genera by the rounded pronotum, without sublateral lines on disc, abdominal sternite VIII without median process in males, and tegmen roundly expanded at apical third (Majer 1989, 1996).

In the original description (Majer 1989), *Danaceothrix* was described monotypically, with *D.murina* Majer, 1989 designated as the type species. Subsequently, another two species were added (Majer 1996). At present, this genus comprises three species, primarily distributed in the Himalayas and neighboring countries (Majer 1989, 1996). In the present study, some specimens of this genus were assembled, and all of them were collected from the Xizang Autonomous Region in Southwest China. Through meticulous examination and identification, we have identified these specimens as two new species, which are described below. Our findings will enhance the understanding of the species diversity among dasytid beetles in the Chinese fauna.

## ﻿Material and methods

In this study, we adhere to the conventional taxonomic classification of dasytid beetles as a separate family, Dasytidae (Majer 1994, 1996; Mayor 2007; Bocakova et al. 2012; Constantin 2015; Miao et al. 2024), rather than regarding them as a subfamily within Melyridae (Gimmel et al. 2019; Gimmel and Mayor 2024). This study primarily focuses on the species descriptions of *Danaceothrix*, despite the ongoing debates regarding its higher classification, which falls outside the scope of this research. The studied specimens are deposited in the Museum of Shanghai Normal University, Shanghai, China (**SHNU**) and the Museum of Hebei University, Baoding, China (**MHBU**).

The specimens were initially soaked in water for softening, followed by the separation of their abdomens. The separated abdomens were then immersed in a 10% sodium hydroxide (NaOH) solution and heated at a constant temperature for several minutes using a metal bath. Once the fat had dissolved, they were transferred to a Nikon SMZ1500 stereo microscope for the dissection of the pygidium, abdominal sternite VIII and genitalia. To facilitate observation, the spiculum gastrale, tegmen, and median lobe were isolated. The ovipositor was stained with hematoxylin. Subsequently, the dissected genitalia were placed on a glass slide with glycerol and photographed using a Leica M205A stereo microscope before being stored in glycerol for preservation. A Canon EOS 80D digital camera was used to capture images of habitus, which were later processed using Helicon Focus ver. 7 software. Adobe Photoshop CC 2019 ver. 20.0.4 was utilized for editing in plate preparation. The body length was measured from the anterior margin of the head to the elytral apices, and the width at the humeri. The terminology of genital segments follows Majer (1994), and that of genitalia follows Gimmel and Mayor (2019).

The distribution map was prepared by ArcMap ver. 10.8 and edited in Photoshop CC 2019 ver. 20.0.4, based on distribution information from the relevant literature (Majer 1989, 1996) and the studied material.

## ﻿Taxonomy

**Class Insecta Linnaeus, 1758**

**Order Coleoptera Linnaeus, 1758**

**Superfamily Cleroidea Latreille, 1802**

**Family Dasytidae Laporte, 1840**

**Subfamily Chaetomalachiinae Majer, 1987**

### ﻿Genus Danaceothrix

Taxon classificationAnimaliaColeopteraDasytidae

Majer, 1989

449E5AD7-B1FF-5B19-A87F-41AA11BD6E1B

#### Updated diagnosis.

Body small-sized, 2.1–4.4 mm in length. Pronotum rounded, without sublateral lines, lateral margins arcuate with sparse crenation (Fig. 2). Tarsi simple, tarsomeres 4 obviously smaller than 3. Abdominal sternite VIII with median process absent in male (Fig. 3A, I), present in female. Tegmen (Fig. 3C, K) subparallel-sided at basal part in ventral view, with submedian dilation distinct or not, roundly expanded at apical third, mostly with a bulb-shaped subapical ring, apex covered with four setae, strongly bent ventrally at basal third in lateral view, the bent part with a more or less distinct median keel on outer surface and a median line on inner surface (Fig. 3D, L); apical limb of median lobe mostly slender, nearly straight or feebly bisinuate ventrally in lateral view (Fig. 3G, O). Internal sac with numerous distinct but short spines (Fig. 3F, G, N, O). Spiculum gastrale (Fig. 3H, P) Y-shaped. Ovipositor (Fig. 4C, F) stout and membranous, gonostylus long and nearly cylindrical, transverse coxital baculus short and arcuate, baculus long and oblique.

#### Included species.

*Danaceothrixmurina* Majer, 1989, *D.glaberrima* Majer, 1996, *D.monilicornis* (Champion, 1922), *D.medogense* sp. nov., and *D.xizangense* sp. nov.

#### Distribution

**Figure 1.**
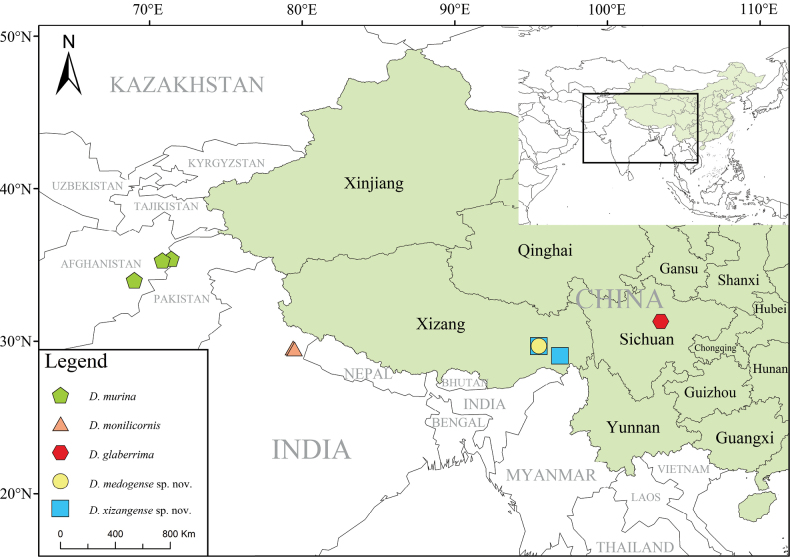
Distribution map of all *Danaceothrix* species.

(Fig. 1). China (Sichuan, Xizang), northwestern India, Afghanistan.

**Figure 2.**
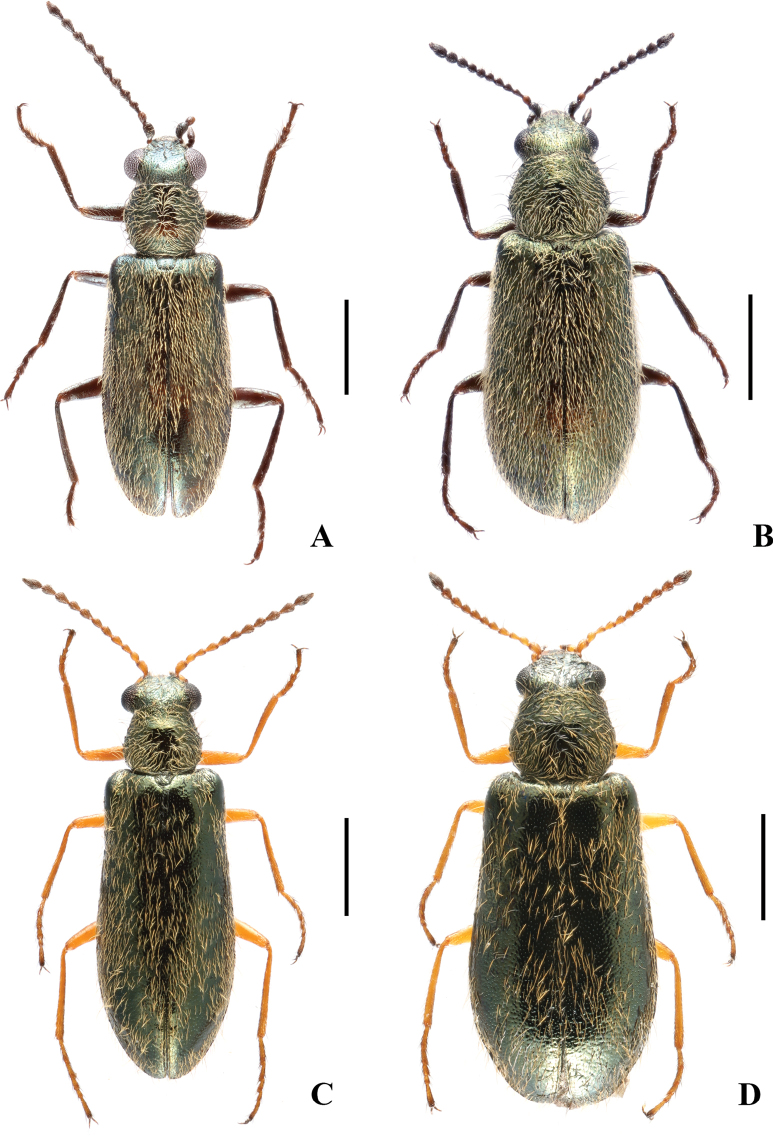
Habitus of *Danaceothrix* species, dorsal view: *D.medogense* sp. nov. (**A, B**), *D.xizangense* sp. nov. (**C, D**). **A, C.** Males; **B, D.** Females. Scale bars: 1.0 mm.

**Figure 3.**
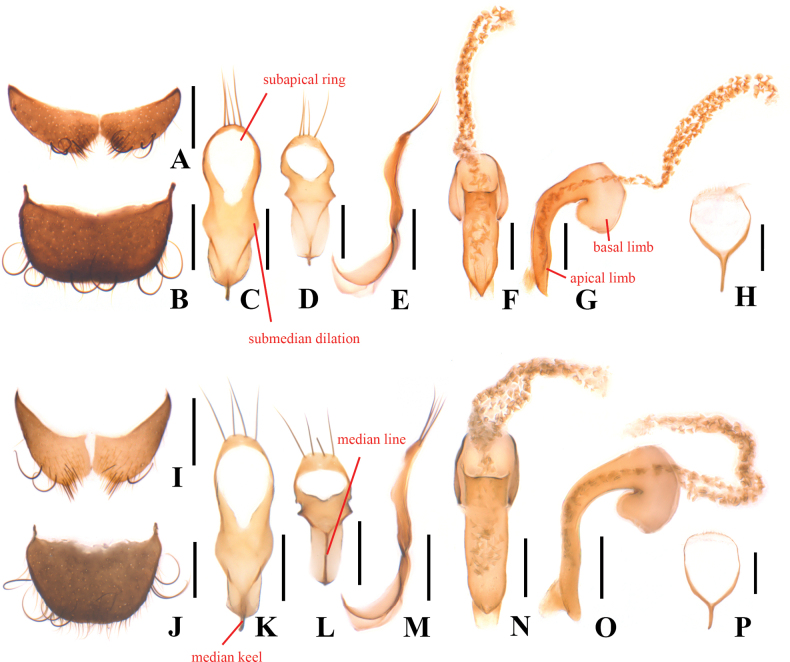
*Danaceothrixmedogense* sp. nov., male (**A–H**), *D.xizangense* sp. nov., male (**I–P**) **A, I.** Abdominal sternite VIII, ventral view; **B, J.** Pygidium, dorsal view; **C, K** Tegmen, ventral view; **D, L.** Tegmen, top view; **E, M.** Tegmen, lateral view; **F, N.** Median lobe, ventral view; **G, O.** Median lobe, lateral view; **H, P.** Spiculum gastrale, ventral view. Scale bars: 0.2 mm.

**Figure 4.**
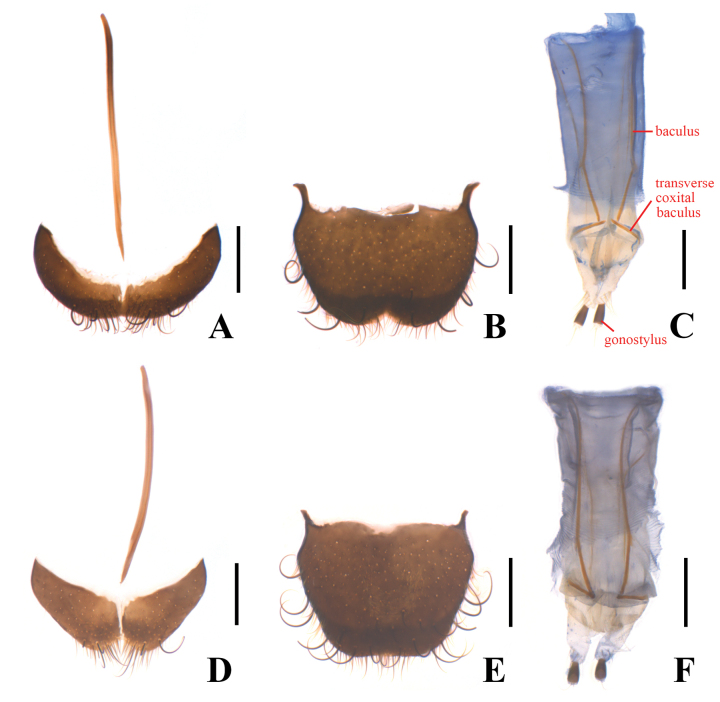
*Danaceothrixmedogense* sp. nov., female (**A–C**), *D.xizangense* sp. nov., female (**D–F**). **A, D.** Abdominal sternite VIII, ventral view; **B, E.** Pygidium, dorsal view; **C, F.** Ovipositor, ventral view. Scale bars: 0.2 mm.

### ﻿ Danaceothrix medogense

Taxon classificationAnimaliaColeopteraDasytidae

Yang & Miao sp. nov.

C07C8A69-FF56-5932-A004-4DE91C60114B

https://zoobank.org/480DD7BC-E00C-4966-9653-FA005D41025F

Figs 1 , 2A, B , 3A–H , 4A–C

#### Diagnosis.

This species can be easily distinguished from all other species of *Danaceothrix* by the shape of the tegmen and median lobe of the aedeagus. Its median lobe looks similar to *D.glaberrima* Majer, 1996, but can be distinguished by the combination of following characters: body black with strong green metallic luster, except for antennomeres 2 brown and trochanters yellowish brown; body surface densely covered with very distinct pubescence (Fig. 2A); pygidium about 1.6 times as wide as long (Fig. 3B); submedian dilation of tegmen distinct and angled (Fig. 3C). Unlike in *D.glaberrima*, the body is dark brown to piceous, tibiae, tarsi and antennomeres 2 are lighter; body almost bare, only pronotum with short setae; pygidium about 3.0 times as wide as long; submedian dilation of tegmen indistinct (Majer 1996: fig. 82).

#### Etymology.

The name of the species is derived from the name of the type locality, Mêdog County, in the Xizang Autonomous Region, China.

#### Type material.

***Holotype*.** China • Xizang: ♂ (MHBU), Nyingchi City, Mêdog County, near 80K, 29°41'09"N, 95°30'10"E, 2330 m, 09.vii.2018, leg. Cheng, Peng & Shen. ***Paratypes*.** China • Xizang: 2 ♀♀ (MHBU), 2 ♀♀ (SHNU), same data as the holotype.

#### Description.

**Male** (Fig. 2A). Body length 4.0 mm, width 1.1 mm.

Body black with strong green metallic luster. Antennae black, except for antennomeres 2 brown, 2–11 without green luster. Legs black, except for trochanters yellowish brown. Body surface densely and shallowly punctate, densely covered with short and recumbent yellow pubescence, and a few long and erect black setae on head and pronotum.

Eyes distinctly prominent, head width across eyes nearly as wide as pronotum. Antennae extending to basal 1/5 length of elytra when inclined, with antennomeres 1 nearly ellipsoidal, 2 globular, 3–10 triangular and longer than wide, 11 fusiform, 2.5 times as long as wide.

Pronotum slightly transverse and 1.2 times as wide as long, widest near basal 2/5, anterior and posterior margins nearly straight, lateral margins arcuate with sparse crenation, anterior and posterior angles widely rounded. Elytra feebly dilated posteriorly, 2.4 times longer than humeral width, 3.2 times longer than pronotum, rounded at apices. Legs slender and simple.

Abdominal sternite VIII (Fig. 3A) strongly transverse and bilobed, each lobe about 2.5 times as wide as long, with antero-lateral angles acute, surface covered with a few long curly black setae and some short, erect setae near middle of posterior margin. Pygidium (Fig. 3B) strongly transverse, 1.6 times wider than long, feebly narrowed posteriorly, slightly arcuate at anterior margin, hardly emarginate in middle of posterior margin, with antero-lateral angles protruding and acute at apices, which are directed posteriorly, surface covered with a few long curly setae along posterior margin.

Aedeagus: tegmen (Fig. 3C–E) with the subapical ring reaching middle, submedian dilation distinctly angled in ventral view; median lobe with apical limb 4.7 times longer than basal width, bisinuate ventrally in lateral view (Fig. 3G), distinctly pointed at apex in ventral view (Fig. 3F). Spiculum gastrale (Fig. 3H) with basal trunk as long as apical branch.

**Female** (Fig. 2B). Similar to male, but body stouter, 3.9–4.1 mm in length, 1.2–1.3 mm in width. Trochanters dark brown. Eyes slightly prominent, head width across eyes narrower than pronotum. Antennae slightly shorter, extending to elytral humeri when inclined. Elytra distinctly dilated across apical third, 2.3 times longer than humeral width, 2.8 times longer than pronotum. Abdominal sternite VIII (Fig. 4A) bilobed, each lobe almost even in width, about 4.0 times as long as wide, with antero-lateral angles acute, the median process very slender and distinctly extending beyond antero-lateral angles. Pygidium (Fig. 4B) shallowly emarginate in middle of anterior margin, large and triangularly emarginate in middle of posterior margin, with antero-lateral angles obviously protruding and acute at apices, which are directed laterally.

#### Distribution

(Fig. 1). China (Xizang).

### ﻿ Danaceothrix xizangense

Taxon classificationAnimaliaColeopteraDasytidae

Yang & Miao sp. nov.

F668B9DB-FB1D-5F30-98EB-6B97E74669AE

https://zoobank.org/7959233B-8907-4D0D-B6BD-E1DFD0BF0BDC

Figs 1 , 2C, D , 3I–P , 4D–F

#### Diagnosis.

This species can be easily distinguished from all other species of *Danaceothrix* by the shape of the tegmen and median lobe of the aedeagus. It looks similar to *D.medogense* sp. nov., but can be distinguished by the combination of following characters: antennae yellow, antennomeres 1–4 darkened at apices, rarely 1 brown, 5–11 more or less darkened; legs yellow, tarsi together with claws more or less darkened at apices; pygidium strongly narrowed posteriorly (Fig. 3J); submedian dilation of tegmen rounded in ventral view (Fig. 3K). Unlike in *D.medogense*, the antennae are black except for antennomeres 2 brown; legs black except for trochanters yellowish brown; pygidium feebly narrowed posteriorly (Fig. 3B); submedian dilation of tegmen angled in ventral view (Fig. 3C).

#### Etymology.

The name of the species is derived from the name of the type locality, Xizang Autonomous Region, China.

#### Type material.

***Holotype*.** China • Xizang: ♂ (MHBU), Nyingchi City, Zayü County, Sangba Village, 29.0423266°N, 96.89045596°E, 2986 m, 28.vii.2024, leg. C. Fang & S. L. Yuan. ***Paratypes*.** China • Xizang: 7 ♂♂ 6 ♀♀ (MHBU), same data as the holotype; 3 ♂♂ 3 ♀♀ (SHNU), Nyingchi City, Mêdog County, near 80K, 29°41'09"N, 95°30'10"E, 2330 m, 09.vii.2018, leg. Cheng, Peng & Shen.

#### Description.

**Male** (Fig. 2C). Body length 3.7–4.1 mm (4.1 mm in holotype), width 1.0–1.2 mm (1.2 mm in holotype).

Body black with strong green metallic luster. Antennae yellow, antennomeres 1–4 darkened at apices, rarely 1 brown, 5–11 more or less darkened. Ultimate maxillary and labial palpomeres yellow and darkened at apices. Legs yellow, tarsi together with claws more or less darkened at apices. Body surface densely and shallowly punctate, densely covered with short and recumbent yellow pubescence, and a few long and erect yellow setae.

Eyes distinctly prominent, head width across eyes feebly wider than pronotum. Antennae extending to basal 1/5 length of elytra when inclined, with antennomeres 1 nearly conical, 2 globular to ellipsoidal, 3–10 triangular and longer than wide, 11 fusiform, 2.2–2.3 times as long as wide.

Pronotum slightly transverse and 1.2 times as wide as long, widest near middle, anterior margin nearly straight, posterior margin arcuate, lateral margins arcuate with sparse crenation, anterior and posterior angles widely rounded. Elytra feebly dilated posteriorly, 2.5–2.7 times longer than humeral width, 3.4–3.9 times longer than pronotum, rounded at apices. Legs slender and simple.

Abdominal sternite VIII (Fig. 3I) strongly transverse and bilobed, each lobe about twice as wide as long, with antero-lateral apical angles sharply protruding, surface covered with a few long curly black setae and some short, erect setae near middle of posterior margin. Pygidium (Fig. 3J) strongly transverse, 1.4 times wider than long, strongly narrowed posteriorly, slightly and roundly emarginate in middle of anterior margin, hardly emarginate in middle of posterior margin, with antero-lateral angles protruding and acute at apices, which are directed posteriorly, surface covered with a few long curly setae along lateral margins.

Aedeagus: tegmen (Fig. 3K–M) with the subapical ring reaching middle, where bearing a pair of small and acute projections on both sides, submedian dilation distinct and rounded in ventral view; median lobe with apical limb 5.7 times longer than basal width, slightly bisinuate ventrally in lateral view (Fig. 3O), indistinctly pointed at apex in ventral view (Fig. 3N). Spiculum gastrale (Fig. 3P) with basal trunk shorter than apical branch.

**Female** (Fig. 2D). Similar to male, but body stouter, 3.8–4.4 mm in length, 1.2–1.4 mm in width. Eyes slightly prominent, head width across eyes narrower than pronotum. Antennae slightly shorter, extending to elytral humeri when inclined. Elytra distinctly dilated across apical third, 2.3 times longer than humeral width, 3.0–3.1 times longer than pronotum. Abdominal sternite VIII (Fig. 4D) bilobed, each lobe distinctly narrowed laterally, about twice as long as wide, with antero-lateral angles acute, the median process very slender and distinctly extending beyond antero-lateral angles. Pygidium (Fig. 4E) shallowly emarginate in middle of anterior margin, nearly straight at posterior margin, with antero-lateral angles obviously protruding and acute at apices, which are directed laterally.

#### Distribution

(Fig. 1). China (Xizang).

##### ﻿Key to the species of *Danaceothrix*

**Table:** 

1	At least some antennomeres wider than long; apical limb of median lobe nearly straight ventrally in lateral view (Majer 1989: fig. 101; Majer 1996: fig. 93)	**2**
–	All antennomeres longer than wide; apical limb of median lobe bisinuate ventrally in lateral view (Fig. 3G, O; Majer 1996: fig. 83)	**3**
2	Body almost bare, very sparsely covered with short hairs; elytra and legs black; elytra feebly dilated posteriorly in males, while distinctly dilated in females; submedian dilation of tegmen rounded in ventral view (Majer 1996: fig. 92). NW India	***D.monilicornis* (Champion, 1922)**
–	Body densely covered with very distinct pubescence; elytra black, rufescent at apical margins; legs testaceous, protarsi together with claws more or less darkened at apices; elytra parallel-sided in males, feebly dilated posteriorly in females; submedian dilation of tegmen distinctly angled in ventral view (Majer 1989: fig. 99). Afghanistan	***D.murina* Majer, 1989**
3	Body almost bare, only pronotum covered with short setae; pygidium about 3.0 times as wide as long; submedian dilation of tegmen indistinct (Majer 1996: fig. 82). China: Sichuan	***D.glaberrima* Majer, 1996**
–	Body densely covered with very distinct pubescence; pygidium about 1.4–1.6 times as wide as long (Fig. 3B, I); submedian dilation of tegmen distinct (Fig. 3C, K)	**4**
4	Antennae black, except for antennomeres 2 brown; legs black, except for trochanters yellowish brown; pygidium feebly narrowed posteriorly (Fig. 3B); submedian dilation of tegmen distinctly angled in ventral view (Fig. 3C). China: Xizang	***D.medogense* sp. nov.**
–	Antennae yellow, antennomeres 1–4 darkened at apices, rarely 1 brown, 5–11 more or less darkened; legs yellow, tarsi together with claws more or less darkened at apices; pygidium strongly narrowed posteriorly (Fig. 3J); submedian dilation of tegmen rounded in ventral view (Fig. 3K). China: Xizang	***D.xizangense* sp. nov.**

## Supplementary Material

XML Treatment for Danaceothrix

XML Treatment for Danaceothrix medogense

XML Treatment for Danaceothrix xizangense

## ﻿Additional information

### Author ORCIDs

Jialin Miao https://orcid.org/0009-0006-6708-6912

Haoyu Liu https://orcid.org/0000-0003-1383-5560

Junbo Tong https://orcid.org/0000-0002-0116-0204

Xingke Yang https://orcid.org/0000-0003-3676-6828

Yuxia Yang https://orcid.org/0000-0002-3118-6659

### Data availability

All of the data that support the findings of this study are available in the main text.
